# Acute Demyelinating Polyneuropathy after Lung Transplantation: Guillain-Barré Syndrome or Tacrolimus Toxicity?

**DOI:** 10.1155/2014/685010

**Published:** 2014-08-11

**Authors:** Nirmal S. Sharma, Keith M. Wille, Charles W. Hoopes, Enrique Diaz-Guzman

**Affiliations:** ^1^Department of Pulmonary & Critical Care, University of Alabama at Birmingham, AL, USA; ^2^Department of Cardiothoracic Surgery, University of Kentucky, Lexington, KY 40506, USA; ^3^UAB ECMO Program, Cardiothoracic Transplant, University of Alabama at Birmingham, 619 19th Street S., Jefferson Tower 1102, Birmingham, AL 35294, USA

## Abstract

Guillain-Barré syndrome (GBS) has been described after solid organ and bone marrow transplantation mostly due to viral infections and possibly calcineurin inhibitors. Incidence after bone marrow transplant is 0.3–0.7%, though incidence in other transplants is not well known. We present the first description of tacrolimus associated GBS in lung transplant recipients in the English language literature. The pathophysiology of tacrolimus-induced polyneuropathy is not known, but some have hypothesized that tacrolimus induces an inflammatory phenomenon by differential effects on T cell subsets. Diagnosis of association may be challenging and requires high index of suspicion. The optimal treatment of GBS-associated with tacrolimus after lung transplantation is unknown, although drug discontinuation may result in improvement in some patients, while some reports suggest that the use of IVIG and/or plasmapheresis may be helpful and safe in organ transplant recipients with severe symptoms.

## 1. Introduction

Guillain-Barré syndrome (GBS) is an autoimmune neurological disorder characterized by acute demyelinating changes of the peripheral nervous system that result in an ascending paralysis and characterized by severe bilateral symmetric weakness of the limbs [1]. The majority of cases are associated with an upper respiratory tract infection, gastrointestinal illness, or recent immunization, but a wide variety of infections and exposures have been described in association with the syndrome [2]. GBS can occur after liver, kidney, heart, lung, and bone marrow transplantation [3]. Although viral infections are the most common attributable cause in organ transplant recipients, calcineurin inhibitors have been postulated as a potential cause of GBS. We describe the course and treatment of two lung transplant recipients diagnosed with GBS occurring in association with the use of tacrolimus.

## 2. Case Report 1

A 68-year-old male with history of COPD presented four months after bilateral lung transplantation complaining of dizziness, generalized weakness, fatigue, and difficulty in ambulating. His immediate postoperative course was uncomplicated and pulmonary function testing was normal after a three-month follow-up. The patient's medications included prednisone (20 mg/day), mycophenolate mofetil (1000 mg twice/day), and tacrolimus (5 mg twice/day). The patient denied a history of upper respiratory infection, diarrhea, recent travel, immunization, or use of new medications prior to the beginning of his symptoms. Physical examination showed normal upper extremities motor strength and symmetric mild reduction of lower extremity strength (3/5) without sensory deficits. Deep tendon reflexes were normal. Tacrolimus level was adjusted to therapeutic target of 10 ng/mL (range 8–12 ng/mL). The average measured tacrolimus level during the prior three months was 10.5 ± 4.1 ng/mL. Microbiology results were negative for cytomegalovirus (CMV) and Epstein Barr virus (EBV) DNA by PCR in the serum. An MRI of the head with contrast was normal. Approximately two weeks later, the patient reported further decline in motor strength and inability to ambulate. Physical exam revealed hypoactive deep tendon reflexes and symmetric bilateral lower extremity weakness (1/5). The patient underwent a lumbar puncture which revealed protein of 76 mg/dL and glucose of 68 mg/dL. Urine, blood, and CSF cultures were negative for bacteria, fungi, and viruses, while a repeat serum CMV DNA by PCR was negative. An MRI of the spine revealed mild degenerative cervical spine disease without evidence of spine compression. Electromyogram (EMG) and nerve conduction studies showed increased latency and decreased amplitude of the left ulnar sensory and left sural sensory and decreased conduction velocity and decreased amplitude of the left tibial motor and left ulnar motor consistent with an acute demyelinating polyneuropathy with axonal changes. At this point, the patient's neurological findings were felt to be secondary to GBS associated with tacrolimus. Tacrolimus was discontinued and the patient was started on cyclosporine. Approximately ten days later, the patient continued with inability to ambulate and developed severe dyspnea. At this point, the patient FEV_1_ decreased by 50% from baseline. The patient was admitted to the ICU and was treated with five sessions of total plasma exchange. Due to the possible association between calcineurin inhibitor use and neurotoxicity, cyclosporine was discontinued and the patient was started on sirolimus. After completion of plasmapheresis, the patient reported a 50% increase in his lower extremity motor strength. Repeat neurophysiologic testing showed improvement in EMG and nerve conduction studies. Over the next several weeks, the patient continued to improve and was discharged from the hospital to complete outpatient rehabilitation. During follow-up, the patient reported episodes of orthostatic hypotension refractory to treatment with fludrocortisones, midodrine, and salt supplementation. These symptoms were attributed to autonomic dysfunction associated with GBS. The symptoms ultimately resolved and the patient was able to regain full physical strength approximately three months later.

## 3. Case Report 2

A 71-year-old male with a history of idiopathic pulmonary fibrosis was seen in clinic eight months after undergoing single lung transplantation with complains of generalized malaise, fatigue, lower extremity weakness, and difficulty in ambulating. His immediate postoperative course was complicated by a fall resulting in a T8 compression fracture not associated with spinal cord injury and requiring nonoperative medical management. Patient's medications during this visit included prednisone (15 mg/day), mycophenolate mofetil (1000 mg twice/day), and tacrolimus (2.5 mg twice/day). Tacrolimus level was adjusted to therapeutic target of 10 ng/mL (range 8–12 ng/mL). The average measured tacrolimus level during the prior six months was 9.4 ± 3.8 ng/mL. Physical examination during this visit showed evidence of decreased motor strength in lower extremities (2/5) and decreased deep tendon reflexes. There were no sensory deficits. An MRI of the spine showed evidence of prior T8 compression fracture but no spinal cord injury. Lumbar puncture revealed CSF glucose of 76 mg/dL and protein of 265 mg/dL. Blood, urine, and CSF cultures were negative for bacterial, viral, or fungal organisms, and serum CMV DNA by PCR was negative. Acetylcholine receptor antibody levels (binding, blocking, and modulating) were not elevated. An EMG revealed polyneuropathy and both sensory and motor axonal demyelination. Nerve conduction studies revealed that motor and sensory conduction studies of the left leg and left arm were significant for absent left sural and ulnar sensory, low amplitude left tibial motor, borderline prolonged latency, and reduced conduction velocity left ulnar motor. Again, the patient's weakness was felt to be consistent with GBS secondary to tacrolimus. The patient was started on plasmapheresis and reported subjective improvement in his lower extremity strength after two sessions of therapy. Tacrolimus was discontinued and sirolimus (4 mg/daily) was added to the patient's immunosuppressive regimen. At the time of his discharge, the patient was able to ambulate with minimal assistance and was referred to outpatient physical therapy to continue his rehabilitation.

## 4. Discussion

We describe two cases of GBS attributed to tacrolimus use after lung transplantation. GBS is an uncommon disease with a worldwide incidence of 1 to 2 cases per 100,000/year [4], whereas the incidence of GBS occurring after solid or bone marrow transplantation is not known. Studies describing complications in bone marrow transplant recipients suggest an incidence of GBS of 0.3–0.7% [5, 6], while a previous review of the literature that included all cases of GBS in transplant recipients found only 30 cases of GBS occurring after solid organ transplantation (13 liver, 6 heart and lung, and 11 kidney transplant recipients). In the majority of these reports, male patients are affected more frequently than females and the onset of symptoms occurred within three months to one year of the transplant [7].

Frequently described as an autoimmune disorder, GBS has been thought to be rare among transplant patients, and immunosuppression has been considered by some as “protective” for the development of GBS [8]. Nevertheless, reports of GBS developing in immunocompromised hosts such as patients with acute leukemia, HIV, and bone marrow transplant recipients demonstrate that GBS may occur despite profound immunosuppression [9].

Among transplant recipients, GBS has been mostly associated with cytomegalovirus infection and recent immunization use [7, 10]. Alternatively, the use of tacrolimus has been postulated by some as a possible and rare cause of GBS in this population. Bronster et al. described a patient developing a subacute form of demyelinating sensorimotor polyneuropathy four months after liver transplantation. The authors concluded that the neurological findings were caused by tacrolimus and the patient improved after drug discontinuation [11]. Similarly, Kaushik et al. described a patient developing Miller-Fischer variant of GBS in a liver transplant recipient. In this report, the authors used the Naranjo probability scale and postulated a probable relation between the clinical manifestations and tacrolimus therapy [12]. The pathophysiology of tacrolimus-induced polyneuropathy is not known, but some have hypothesized that tacrolimus induces an inflammatory phenomenon by differential effects on T cell subsets [13].

To our knowledge, our report is the first that suggests an association between GBS and tacrolimus in lung transplantation. GBS occurring after lung transplantation has rarely been reported in the literature. Falk et al. described a lung transplant recipient developing GBS in association with cyclosporine [14]. Interestingly, our first case presented with clinical features of autonomic dysfunction similar to those described by Falk et al. in their report.

The differential diagnosis of GBS after organ transplantation in patients receiving tacrolimus includes peripheral sensorimotor polyneuropathy and chronic inflammatory demyelinating polyneuropathy (CIDP). The association between tacrolimus and peripheral mononeuropathy or polyneuropathy is well described in the literature [15]. The pathophysiology of tacrolimus-neurotoxicity is not well studied, although most cases are described in association with high systemic levels of the drug [16, 17]. Although nerve conduction studies in patients with tacrolimus polyneuropathy may also show evidence of demyelinating changes, the symmetric ascending presentation and the absence of a history of elevated tacrolimus levels favor the diagnosis of GBS over tacrolimus polyneuropathy in our patients.

CIDP has been rarely reported affecting transplant patients. Nevertheless, a recent prospective study found an incidence of 0.6% among 1557 solid organ transplant recipients suggesting a higher prevalence in transplant recipients compared to the general population [18]. CIDP shares many features with GBS and therefore establishing the correct diagnosis may be sometimes difficult. The latter is frequently described as an acute clinical syndrome, whereas symptoms in CIDP tend to progress slowly over eight weeks or several months. In addition, patients with GBS frequently have prominent motor involvement with relatively few sensory findings, and autonomic dysfunction is uncommon in patients with CIDP [19].

The optimal treatment of acute or chronic demyelinating polyneuropathy associated with tacrolimus in transplant recipients is unknown. Several reports of liver and renal transplant patients treated with IVIG and/or plasmapheresis show that these therapies are successful and result in neurological improvement, although other reports of tacrolimus induced polyneuropathy also suggest that discontinuation of the drug without further therapy results in similar improvement [20]. Labate et al. described a heart transplant recipient with clinical features suggestive of CIDP with improvement of clinical findings and resolution of demyelinating features three months after discontinuation of tacrolimus [21]. In our first case, discontinuation of tacrolimus did not result in improvement of the symptoms, although due to severity of the weakness we initiated plasma exchange soon after tacrolimus discontinuation. While it is possible that both patients could have improved clinically without further therapy after drug discontinuation, we elected for plasma exchange therapy due to the severity of their neurologic derangements.

We conclude that GBS after organ transplantation is rare. Nevertheless, recent reports suggest that chronic demyelinating polyneuropathy may be more common in transplant recipients than in the general population [18]. In addition, it is likely that the disease is underrecognized and many patients may be erroneously diagnosed with physical deconditioning or peripheral polyneuropathy associated with comorbid conditions (i.e., diabetes, etc.). If undiagnosed, these conditions may lead into severe disability; therefore, we feel that transplant physicians must be aware of the possible association between tacrolimus and GBS or CIDP. The optimal treatment of GBS associated with tacrolimus after lung transplantation is unknown, although drug discontinuation may result in improvement in some patients, while most reports suggest that the use of IVIG and/or plasmapheresis may be helpful and safe in organ transplant recipients with severe symptoms.

## Abbreviations

CIDP: Chronic inflammatory demyelinating polyneuropathy
CMV: Cytomegalovirus
CSF: Cerebrospinal fluid
COPD: Chronic obstructive pulmonary disease
EBV: Epstein Barr virus
EMG: Electromyogram
FEV_1_: Forced expiratory volume in one second
GBS: Guillain-Barré syndrome
PCR: Polymerase chain reaction.
